# MFCAMNet: predicting miRNA-disease associations by multi-feature cascade attention mechanism network

**DOI:** 10.1186/s12859-026-06536-7

**Published:** 2026-07-24

**Authors:** Yuanxing Wang, Chen Yang, Yucheng Zhang

**Affiliations:** 1 Department of Computer Science, Xijing University, 1 Xijing Road, Xi’an, 710123 Shaanxi China; 2 Department of Computer Science, Xi’an University of Technology, 5 Jinhua South Road, Xi’an, 710048 Shaanxi China

**Keywords:** MiRNA-disease association prediction, MFCAMNet, Multi-source feature extraction, Multi-feature fusion, Self-attention mechanism

## Abstract

Drive by the rapid envolution of deep-learning techniques, a large body of biological experiments bas has uncovered extensive associations between microRNAs (miRNAs) and complex human diseases, hig- hlighting the pivotal roles of miRNAs in pathogenesis. Elucidating these associations is essential for understanding disease mechanisms and developing preventive strategies. Traditional wet-lab validati- on, however, is notoriously labor- and resource-intensive, creating an urgent demand for efficient computational tools that can prioritize the most promising miRNA–disease candidates. Existing predictors predominantly rely on a single category of handcrafted features, thereby overlooking the complementary information embedded in multiple, heterogeneous data sources. Although a few recent attempts integrate diverse features, they usually exploit only a limited subset and fail to capture the intricate, non-linear relationships among them. To address these limitations, we propose MFCAMNet, a Multi-Feature fusion and Cross-Self-Attention model for MiRNA–Disease association prediction. Firstly, we construct multiple similarity matrices and employ two independent autoencoders with multi-source feature attention to obtain deep features of miRNA and disease to extract the inherent relationships between multiple features. Secondly, the proposed model employs element-level addition, element-level multiplication, and concatenation operations to generate miRNA-disease pair features with rich information. Finally, we use the encoder structure of the transformer to fuse the three deep features and predict all potential miRNA disease associations. We conducted comprehensive evaluations on the public HMDD v2.0 and HMDD v3.2 benchmark datasets. MFCAMNet achieved average AUCs of 0.9455 and 0.9420 under 5-fold and 10-fold cross-validation on HMDD v2.0, respectively, and an AUC of 0.9578 under 5-fold cross-validation on HMDD v3.2, outperforming state-of-the-art competitors. Case studies on breast, esophageal, and lung cancers further corroborate the reliability and practical utility of the proposed method.

## Introduction

Microrna(miRNA) is a class of endogenous small noncoding RNA with a length of approximately 22 nucleotides that plays essential regulatory roles in various biological processes, including inflammation, cell cycle regulation, stress response, differentiation, apoptosis, and migration [1, 2]. Since the discovery of the first miRNA these molecules have moved to the forefront of biomedical research; abundant evidence now links miRNA dysregulation to the initiation and progression of a wide spectrum of human disorders. The miR-17-92 cluster, for example, is implicated in colorectal carcinoma, B-cell lymphoma, gastric cancer and small-cell lung cancer [3–6]; miR-195 and miR-497 suppress proliferation and invasion of breast-cancer cells and their expression inversely correlates with tumour grade, positioning them as promising diagnostic and therapeutic targets [7]. In oesophageal malignancies miR-19b abundance positively correlates with tumour size, whereas miR-17a over-expression is associated with lymph-node metastasis and advanced stage [8], and miR-155 is frequently up-regulated in lung and other solid cancers [9]. Conventional experimental validation is costly and time-consuming; fortunately, the rapid expansion of bioinformatics and high-performance computing has been accompanied by the release of comprehensive public repositories that curate miRNA- and disease-related data [10–15]. Exploiting these resources, together with state-of-the-art algorithms, enables large-scale in-silico prediction and prioritisation of miRNA–disease associations. The increasing penetration of deep learning into computational biology [16, 17] has given rise to two main families of predictive models: (i) classical architectures such as fully connected, convolutional or recurrent neural networks, and (ii) graph-neural-network approaches built upon graph convolutional networks.

In the mining of miRNA and complex-disease features and association prediction, traditional machine-learning and deep-learning approaches have complementary emphases and are often used synergistically. Classical machine-learning models—such as support-vector machines (SVM), random forests (RF) and gradient-boosting decision trees (GBDT)—usually serve as the core predictor, frequently coupled with k-means clustering to balance positive and negative samples or to select salient features. Deep-learning methods exploit fully connected networks, convolutional neural networks, recurrent neural networks, as well as special architectures like stacked auto-encoders and variational auto-encoders, to extract dense, high-dimensional representations from multi-source data (e.g., disease semantic similarity, miRNA functional similarity, heterogeneous interaction networks), thereby providing richer information for subsequent association inference. For example, Shen et al. [18] proposed CMFMDA, a collaborative-matrix-factorization model that learns a shared latent vector space for miRNAs and diseases and reconstructs the unknown association matrix via low-rank factorization for efficient prediction. Fu et al. [19] presented DeepMDA, a deep ensemble framework that uses stacked auto-encoders to capture high-order features from miRNA and disease similarity matrices and predicts association probabilities in an end-to-end manner. Zhang et al. [20] developed PCACFMDA, which fuses multi-dimensional similarity features, employs PCA to remove redundant dimensions, and feeds the compressed representations into an optimized cascade forest for accurate association scoring. Zhou et al. [21] introduced GBDT-LR: k-means clustering first filters reliable negative samples from unlabeled miRNA–disease pairs, GBDT then automatically extracts discriminative feature combinations, and finally logistic regression outputs the association score. Similarly, Zhao et al. [22] adopted k-means-based negative-sample sampling in the ABMDA framework to balance the training set and improve prediction performance.

Recently, deep-learning-based architectures have also been extensively employed to infer miRNA–complex-disease associations. For instance, Ha et al. [23] argued that conventional approaches inadequately model the non-linear interplay between miRNAs and diseases and suffer from low accuracy under data sparsity; they therefore proposed NCMD, a Node2vec-based neural collaborative filtering framework that first embeds miRNA and disease networks into low-dimensional vectors via Node2vec and then fuses generalized matrix factorization (GMF) and a multi-layer perceptron (MLP) to capture linear and non-linear interactions, respectively, for final association scoring. Building upon this line of research, Ha et al. [24] recently proposed GCNCF, a graph convolutional network with neural collaborative filtering that integrates GCN-based node embedding with NCF to simultaneously capture network structural information and complex miRNA-disease interactions. Subsequently, Ha et al. [25] proposed DWMDA, a DeepWalk-based graph embedding method that employs random walks and skip-gram models to extract low-dimensional node representations from miRNA and disease networks, followed by a deep neural network for association prediction. Lou et al. [26] noticed that standard graph convolutional networks (GCNs) exploit only first-order neighbourhood information and hence fail to encode high-order relationships; they presented MINIMDA, a hybrid-neighbourhood model that simultaneously aggregates 0–k-hop neighbourhood information within a unified multi-modal graph via refined graph convolutional layers to learn deep embeddings for miRNAs and diseases, followed by an MLP for association prediction. Echoing this, Ata et al.[27] proposed a collaborative multi-view network embedding framework, which can effectively fuse structural and attribute information in multiple views and enhance the embedding quality through collaborative learning among views. Ai et al. [28] devised an end-to-end framework named MHDMF that employs GCN to integrate multi-source information about miRNAs, genes and diseases, reconstructs the miRNA–disease scoring matrix, and subsequently applies deep matrix factorization (DMF) to uncover latent associations. Kim and Ha [?] recently proposed GMFLDA, which adapts this architecture for lncRNA-disease association prediction, achieving competitive performance under both leave-one-out and five-fold cross-validation frameworks. Prior to this, Ha [29] proposed EMFLDA, an lncRNA expression profile-based matrix factorization framework that incorporates expression data as implicit feedback to address the high false-negative rate problem in lncRNA-disease association prediction. Ji et al. [30] addressed the absence of reliable negative samples and the resulting training difficulties of traditional supervised methods by introducing AEMDA, an autoencoder-based model that first uses ridge regression to derive high-dimensional vector representations from integrated miRNA and disease similarity profiles and then feeds these vectors into a deep autoencoder whose reconstruction error serves as the association strength, enabling prediction without explicit negatives. Wang et al. [31] pre-trained a stacked autoencoder (SAE) in an unsupervised manner on all available miRNA–disease pairs, subsequently introduced positive and negative samples for supervised fine-tuning, and finally predicted novel associations by combining reconstruction error with a softmax classifier.Beyond RNA-disease association prediction, similar deep learning architectures have been successfully applied to drug-target interaction prediction. Ha [32] recently proposed the SVDTI framework, which employs a stacked variational autoencoder. This approach processes drug SMILES representations and protein sequences via LSTM networks, subsequently utilizing neural collaborative filtering for interaction prediction.

Despite the above-mentioned progress in miRNA–disease association prediction, two salient limitations remain. First, prevailing deep-learning pipelines still adopt a fixed fusion recipe—either simple concatenation or element-wise addition—without a systematic comparison among alternative strategies; for instance, AEMDA merely concatenates the high-dimensional miRNA and disease vectors before feeding them into the auto-encoder, leaving the optimal combination unexplored. Second, some studies rely on a single data source, whereas others [19, 31] incorporate multiple omics but exploit them only superficially or in isolation, failing to excavate their inherent complementarity and deep cross-modal correlations, thereby constraining further performance gains.

To remedy the aforementioned shortcomings, we present MFCAMNet, a novel computational framework that unites multi-feature processing with self-attention for miRNA–disease association prediction. The rationale is rooted in prior evidence: MINIMDA [33] and AMHMDA [34] improved accuracy by exploiting multi-source miRNA and disease representations; DDIMDL [35] achieved precise drug–drug interaction (DDI) event forecasting through integrating heterogeneous drug features; and MDFSA-DDI [36] further advanced DDI prediction by deepening this multi-source fusion philosophy. Building on these insights, MFCAMNet learns multimodal representations of miRNAs and diseases from diverse data sources and then infers their latent associations. Its innovative contributions are summarized as follows.

- We propose the MFCAMNet architecture. The architecture firstly achieves efficient miRNA and disease feature extraction through the multi-source feature attention mechanism, and then performs element-level addition, element-level multiplication and feature splicing operations in parallel to construct an interaction-rich miRNA-disease pair representation. Comparative experiments systematically analyse the performance impact of each fusion strategy, providing empirical guidance for choosing the optimal solution in miRNA-disease association prediction.
- We designed a novel multi-source similarity fusion network to capture the intrinsic properties and association patterns of miRNAs and diseases. MFCAMNet integrates four complementary similarity dimensions, namely, miRNA functional similarity and miRNA Gaussian similarity, disease semantic similarity, and Gaussian interaction spectral kernel similarity, as well as ensemble similarity matrices constructed for miRNAs and diseases, respectively. By deeply fusing these heterogeneous data, a solid foundation for accurate representation learning is laid.
- we propose and systematically verify for the first time the effectiveness of a parallelised multilevel feature fusion strategy for enhancing the prediction performance of miRNA-disease associations. Unlike the single fusion approach commonly used in existing methods, we innovatively introduced three operations, namely element-by-element addition, element-by-element multiplication and feature splicing, to construct miRNA-disease pair representations in parallel, thus capturing multiple interaction modes, such as linear complementarity, nonlinear interaction and original information retention. By designing and comparing seven different fusion combinations, this study establishes for the first time that the ‘addition-multiplication-splicing’ strategy can produce optimal performance, providing a novel design paradigm for the selection of feature fusion schemes in this field.
- Comprehensive performance evaluation and reliability verification: systematic experiments on the public HMDDv2.0 and HMDDv3.2 datasets demonstrate that MFCAMNet outperforms competing methods in terms of both AUC and AUPR. Furthermore, three disease-specific case studies corroborate the robustness and practical utility of the proposed framework.

The rest of this paper is organized as follows. Section II illustrates the details of the proposed framework. The experiment and analysis are allocated in Section III. Finally, conclusions are drawn in Section IV.

## Materials and methods

### Human miRNA-disease association

The Human microRNA Disease Database (HMDD) is the recognized gold-standard benchmark in miRNA–disease association mining. Both mainstream releases—HMDD v2.0 [14] and HMDD v3.2 [12]—are freely available from the official portal https://www.cuilab.cn/hmdd. HMDD v2.0 contains 5 430 experimentally verified human miRNA–disease associations covering 495 miRNAs and 383 diseases; the newest release, HMDD v3.2, expands the collection to 8968 associations among 788 miRNAs and 374 diseases, offering a substantial increase in scale and coverage. From these data we construct a binary adjacency matrix $A \in {R^{{n_m} \times {n_d}}}$, where ${n_m}$ and ${n_d}$ denote the numbers of miRNAs and diseases, respectively. If and only if there is an experimentally supported association between miRNA *i* and *j* disease , the matrix element ${A_{ij}} = 1$; otherwise, ${A_{ij}} = 0$. This matrix provides a unified and extensible initial topology for heterogeneous networks and is widely adopted for algorithmic performance evaluation and comparative analysis. Table 1 shows the statistics for these two datasets.

**Table 1:** The statistics for dataset

Dataset	miRNA	disease	Assocaition
HMDD v2.0	495	383	5430
HMDD v3.2	788	374	8968

According to the hypothesis, functionally similar miRNAs are usually associated with similar disease and vice versa [37]. We calculated the functional similarity between miRNAs using the MISIM dataset [38] and obtained the similarity matrix $MFS \in {R^{{n_m} \times {n_m}}}$.

### Disease semantic similarity

Disease semantic similarity is commonly quantified on the basis of Medical Subject Headings (MeSH) descriptors, whose hierarchical structure can be formalized as a directed acyclic graph (DAG). In this DAG, nodes correspond to specific disease entities and directed edges denote parent–child or broader–narrower semantic relationships. This computational paradigm has been widely adopted in previous studies and has crystallized into two mainstream strategies. The first family of methods [37] rests on the assumption that the higher the overlap of the common ancestor sets of two diseases within the DAG, the greater their semantic similarity score. Concretely, by counting the shared ancestor nodes and weighting their contributions according to hierarchical level, one can derive a quantitative semantic similarity measure. The detailed computation proceeds as follows.

1 $$\begin{aligned} D 1_A(\textrm{i}, \textrm{j})=\left\{ \begin{array}{cc} 1 & , \textrm{i}=j \\ \max \left\{ \omega * D 1_A(\textrm{i}, t)\right\} & , i \ne j \end{array}\right. \end{aligned}$$

where *ω * is a hyperparameter, and the value is set to 0.5. Assuming that $Anc({d_i})$ represents the set of nodes containing its own nodes and ancestor nodes, the semantic similarity of the disease pair $({d_i},{d_j})$ can be calculated by the following formula.

2 $$\begin{aligned} D{S_1} = \frac{{\sum \nolimits _{\mathrm{{t}} \in Anc(i) \cap Anc(j)} {\left( {D{1_A}\left( {i,t} \right) + D{1_A}(j,t)} \right) } }}{{\sum \nolimits _{\mathrm{{t}} \in Anc(i)} {D{1_A}(i,t)} + \sum \nolimits _{\mathrm{{t}} \in Anc(j)} {D{1_A}(j,t)} }} \end{aligned}$$

The second calculation method employs the improved disease semantic similarity strategy proposed by Xuan et al. [39], which was specifically designed to address the limitations of the first approach by integrating both disease semantic information content and phenotypic similarity. Its core logic is to enhance the accuracy of semantic similarity assessment by quantifying the specificity of disease terms and the phenotypic associations between diseases. The calculation is as follows:

3 $$\begin{aligned} D{2_A}(i,j) = - log\left( {\frac{{Num({d_j})}}{{Num({d_{all}})}}} \right) \end{aligned}$$

Here, $$Num({d_j}) $$ denotes the occurrence frequency of a specific disease $$d_j$$ in the MeSH database, and $$Num({d_all}) $$ represents the total number of disease entries in the database; based on this, the semantic similarity between diseases $$d_i$$ and $$d_j$$ can be re-estimated using the following expression:

4 $$\begin{aligned} D{S_2} = \frac{{\sum \nolimits _{\mathrm{{t}} \in Anc(i) \cap Anc(j)} {\left( {D{2_A}\left( {i,t} \right) + D{2_A}(j,t)} \right) } }}{{\sum \nolimits _{\mathrm{{t}} \in Anc(i)} {D{2_A}(i,t)} + \sum \nolimits _{\mathrm{{t}} \in Anc(j)} {D{2_A}(j,t)} }} \end{aligned}$$

After obtaining the semantic similarity of these two diseases, the final semantic similarity matrix can be obtained by the fol- lowing equation:

5 $$\begin{aligned} DSS = \frac{{D{S_1}({d_i},{d_j}) + D{S_2}({d_i},{d_j})}}{2} \end{aligned}$$

### Gaussian interaction profile (GIP) Kernel similarity for miRNAs and diseases

**Fig. 1:**
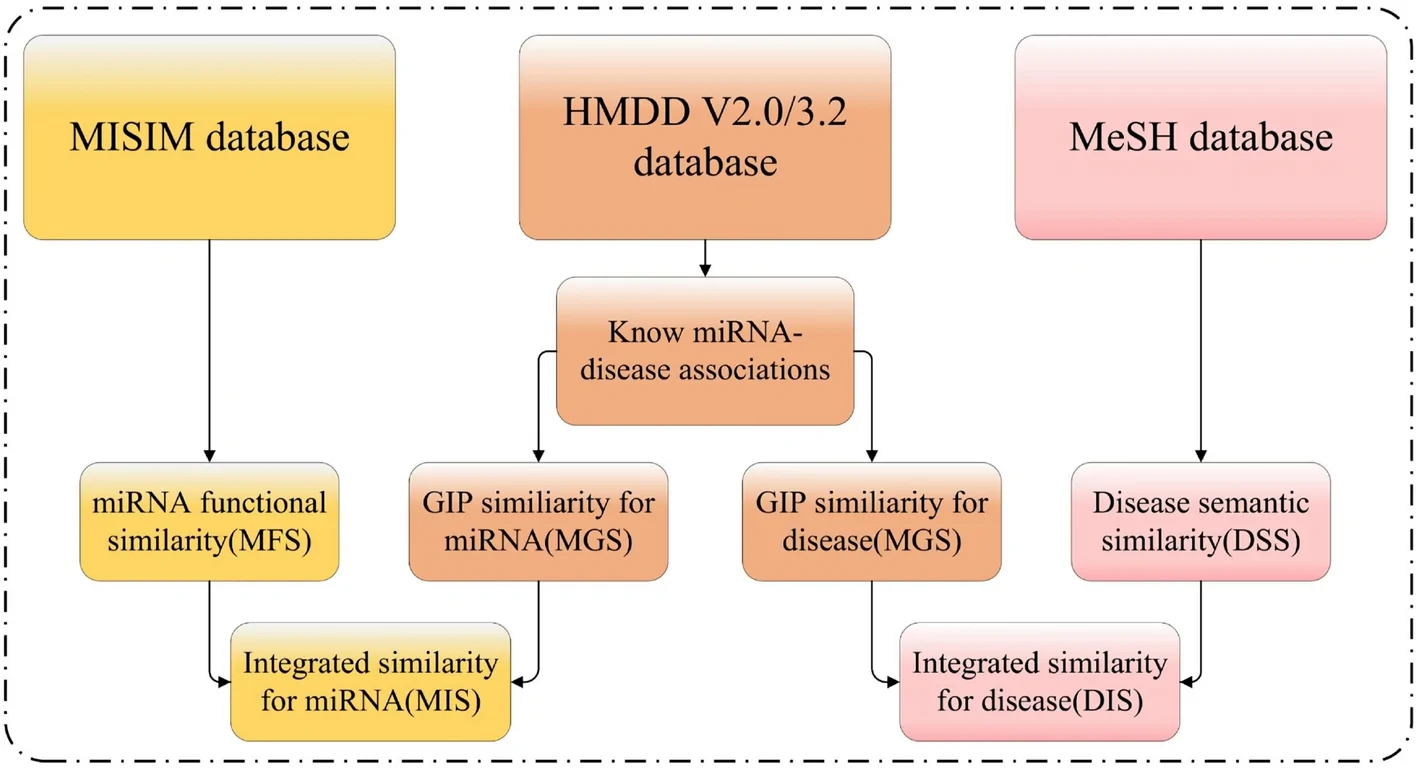
Computing multiple similarity matrices based on multiple data sources about miRNAs and diseases

In previous studies, GIP kernel similarity has been considered an auxiliary of feature for complementary information. It is based on the assumption that the semantics of miRNA associated with functionally similar diseases are also similar. The higher the kernel similarity of GIP between diseases, the higher the similarity of their associated miRNA. We calculated the miRNA GIP similarity based on previous research [33], defined as follows:

6 $$\begin{aligned} & {\gamma _m} = \frac{{{{\gamma '}_m}}}{{\frac{1}{{{n_m}}}\sum \nolimits _{k = 1}^{{n_m}} {{{\left\| {IP({m_k})} \right\| }^2}} }} \end{aligned}$$

7 $$\begin{aligned} & MGS({m_i},{m_j}) = \exp \left( { - {\gamma _m}{{\left\| {IP({m_i}) - IP({m_j})} \right\| }^2}} \right) \end{aligned}$$

According to the previous study, we set ${\gamma '_m}$ equal to 1, and $n_m$ represents the number of known miRNAs. $IP({m_i})$ is denoted as ith column in the miRNA-disease association matrix. Similarly, we can also calculate disease GIP similarity as an informative complement to disease semantic similarity, and the calculation process is as follows:

8 $$\begin{aligned} & {\gamma _d} = \frac{{{{\gamma '}_d}}}{{\frac{1}{{{n_d}}}\sum \nolimits _{k = 1}^{{n_d}} {{{\left\| {IP({d_k})} \right\| }^2}} }} \end{aligned}$$

9 $$\begin{aligned} & MGS({d_i},{d_j}) = \exp \left( { - {\gamma _d}{{\left\| {IP({d_i}) - IP({d_j})} \right\| }^2}} \right) \end{aligned}$$

Similarly, we set ${\gamma '_d}$ equal to 1 while $n_d$ represents the number of known diseases. $IP({d_i})$ is denoted as ith row in the miRNA-disease association matrix. Following the previous study [28], we calculate the miRNA integration similarity and disease integration similarity, and these two types of similarities were calculated as follows:

10 $$\begin{aligned} & \operatorname {MIS}\left( \textrm{m}_i, \mathrm {~m}_j\right) = {\left\{ \begin{array}{ll}\operatorname {MFS}\left( \textrm{m}_i, \mathrm {~m}_j\right) , & \textrm{m}_i \text{ sim } \textrm{m}_j \\ \operatorname {MGS}\left( \textrm{m}_i, \mathrm {~m}_j\right) , & \text{ otherwise } \end{array}\right. } \end{aligned}$$

11 $$\begin{aligned} & \operatorname {DIS}\left( \textrm{d}_i, \mathrm {~d}_j\right) = {\left\{ \begin{array}{ll}\operatorname {DGS}\left( \textrm{d}_i, \mathrm {~d}_j\right) , & \textrm{d}_i \text{ sim } \textrm{d}_j \\ \operatorname {DGS}\left( \textrm{d}_i, \mathrm {~d}_j\right) , & \text{ otherwise } \end{array}\right. } \end{aligned}$$

where the above-mentioned formula calculates $MFS({m_i},{m_j})$, $MGS({m_i},{m_j})$, $DSS({d_i},{d_j})$, and $DGS({d_i},{d_j})$.

### MFCAMNet

**Fig. 2:**
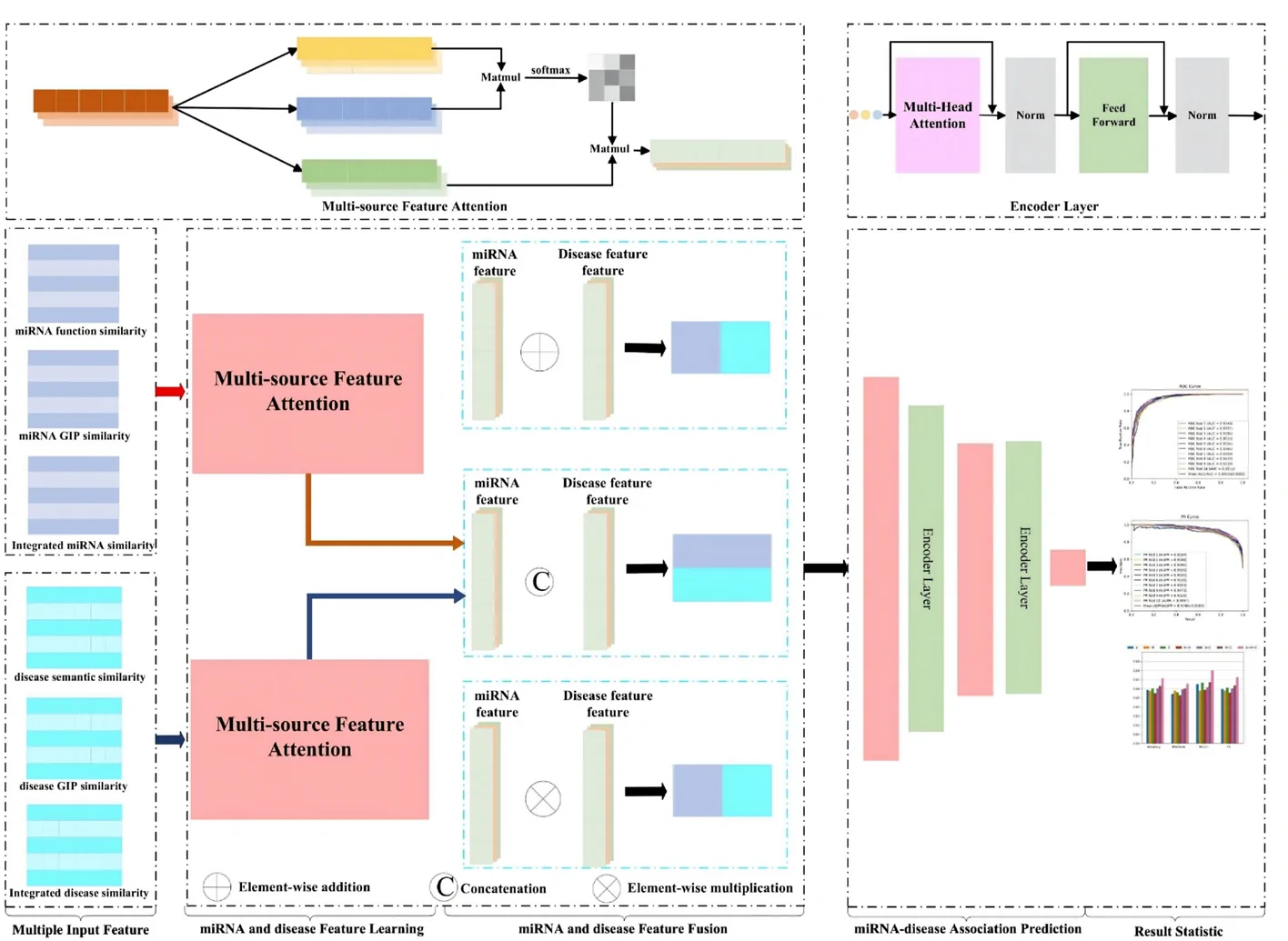
The overall architecture of the proposed MFCAMNet

To reduce the noise and redundancy of the original input features, we utilize an autoencoder with multi-source feature attention to mine the intrinsic distribution of the multi-source features while reducing the dimensions of the multi-source features. Subsequently, the three feature fusion strategies, namely element-level addition, element-level multiplication, and concatenation operations, are used to fuse miRNA and disease features and send them into the classifier for prediction. As shown in Fig. 2, MFCAMNet consists of three mainly stages: miRNA and disease feature learning, miRNA and disease feature fusion, and miRNA-disease pair association prediction. Multiple sources of miRNA and disease features are first fed into autoencoders specific to each class. Both autoencoders receive multiple high-dimensional input features and simultaneously output low-dimensional latent features. The only difference is the type of input features, which allow the miRNA and disease features to be independently learned. It is essential to note that the autoencoder uses Multi-source Feature Attention to weigh and sum the features from multiple sources to help identify the portion of features that are helpful for prediction. Then, we fuse miRNA and disease features using three different methods to obtain features from different perspectives. The final predictions are made using the encoder structure from Transformer, which integrates multi-head attention, to learn the miRNA-disease pair features. Next, we will elaborate on these three stages.

#### Multiple input feature

Multiple source features can provide deep learning models with diverse information from various perspectives to improve the accuracy of miRNA-disease association prediction compared to work using only a single feature. Other miRNA-disease association prediction models that used multiple sources of feature fusion strategy in a simple way and did not further examine the intrinsic distribution among multiple features. In our research, first construct multiple input similarity matrices, as depicted in Fig. 1.

The construction of multiple similarity matrices is motivated by the complementary information each source provides. miRNA functional similarity captures shared biological pathways and regulatory roles; disease semantic similarity encodes hierarchical relationships from medical ontologies; Gaussian interaction profile kernel similarity reflects topological association patterns derived from known miRNA–disease networks; and integrated similarity synthesizes these aspects to mitigate data sparsity and provide a comprehensive representation. Together, these matrices enable a multi-faceted characterization of miRNAs and diseases, allowing the model to leverage both biological semantics and network topology for more robust association inference.

The constructed similarity matrices originated from different bioinformatics repositories and computational paradigms, with significant heterogeneity in the range of values, sparsity and distribution patterns. To ensure the fairness and validity of feature fusion, all input matrices are preprocessed with z-score normalisation to have zero mean and unit variance, thus eliminating scale differences. More importantly, the subsequent MSFA module, by learning dynamic weights for each data source, is able to assign higher weights to sources with more stable distributions and richer predictive information, and vice versa to suppress noise effects. This two-layer mechanism of static normalisation and dynamic attention enables the model to robustly handle heterogeneous inputs.

#### miRNA and disease feature learning

In the miRNA and disease feature learning phases, we use two independent multi-source feature-attentive self-encoders to learn multiple input features for miRNA and disease, respectively. For miRNA, we use three similarity matrices: functional similarity matrix $x_{MFS}$, GIP similarity matrix $x_{MGS}$, and integration similarity matrix $x_{MIS}$. for disease, we use disease semantic similarity matrix $x_{DSS}$, disease GIP similarity matrix $x_{DGS}$, and disease integration similarity matrix $x_{DIS}$.

First, for miRNA feature learning, we input the three similarity matrices into the Multi-source Feature Attention (MSFA) module to obtain an attention-weighted feature representation. The calculation process is as follows:

12 $$\begin{aligned} {f_{mfs}},{f_{mgs}},{f_{mis}} = MSFA({x_{MFS}},{x_{MGS}},{x_{MIS}}) \end{aligned}$$

where $f_{mfs}$, $f_{mgs}$, $f_{mis}$ are weighted features; the MSFA module fuses these multi-source features by assigning different weights to each matrix through the attention mechanism.The weighted features are then fed into a multilayer perceptron for dimensionality reduction to obtain low-dimensional feature representations $h_{mfs}$, $h_{mgs}$, $h_{mis}$. Meanwhile, the weighted features are input to another multilayer perceptron for reconstruction to obtain the reconstructed features $O_{mfs}$, $O_{mgs}$, $O_{mis}$ for subsequent reconstruction loss calculation.The calculation process is as follows:

13 $$\begin{aligned} & h_{mfs},h_{mgs},h_{mis}=MLP_{\theta _1}\left( f_{mfs},f_{mgs},f_{mis} \right) \end{aligned}$$

14 $$\begin{aligned} & O_{mfs},O_{mgs},O_{mis}=MLP_{\gamma _1}\left( f_{mfs},f_{mgs},f_{mis} \right) \end{aligned}$$

where ${\theta _1}$ is the parameter for the downscaled MLP; here, ${\gamma _1}$ is the parameter for the reconstructed MLP.Finally, the downscaled features $h_{mfs}$, $h_{mgs}$, $h_{mis}$ are spliced together to form the final low-dimensional feature representation of miRNA $F_{miRNA}$.

15 $$\begin{aligned} {F_{miRNA}} = Concat({h_{mfs}},{h_{mgs}},{h_{mis}}) \end{aligned}$$

Similarly, for the learning of disease features, we followed the same learning steps as for miRNA features. The three disease similarity matrices are attentively weighted by the *MSFA* module to obtain the weighted feature representations $d_{dss}$, $d_{dgs}$, $d_{dis}$.

16 $$\begin{aligned} {d_{dss}},{d_{dgs}},{d_{dis}} = MSFA({x_{DSS}},{x_{DGS}},{x_{DIS}}) \end{aligned}$$

where $x_{DSS}$, $x_{DGS}$, $x_{DIS}$ are disease semantic similarity matrix, disease GIP similarity matrix, and disease integration similarity matrix respectively.Then, the weighted disease features are input to the downscaled MLP to obtain the low-dimensional feature representations $h_{dss}$, $h_{dgs}$, $h_{dis}$. Meanwhile, the weighted disease features are inputted into the reconstruction MLP to obtain the reconstructed features $O_{dss}$, $O_{dgs}$, $O_{dis}$.

17 $$\begin{aligned} & h_{dss},h_{dgs},h_{dis}=MLP_{\theta _2}\left( d_{dss},d_{dgs},d_{dis} \right) \end{aligned}$$

18 $$\begin{aligned} & O_{dss},O_{dgs},O_{dis}=MLP_{\gamma _2}\left( h_{dss},h_{dgs},h_{dis} \right) \end{aligned}$$

where ${\theta _2}$ is the parameter of disease feature downscaling MLP; ${\gamma _2}$ is the parameter of disease feature reconstruction MLP. Finally, the downscaled disease features $h_{dss}$, $h_{dgs}$, $h_{dis}$ are stitched together to form the final low-dimensional feature representation of the disease $F_{disease}$.

19 $$\begin{aligned} F_{disease}=Concat\left( h_{dss},h_{dgs},h_{dis} \right) \end{aligned}$$

Through the above steps, we obtained the low-dimensional feature representations $F_{miRNA}$ and $F_{disease}$ for miRNAs and diseases, which will be used for subsequent feature fusion and association prediction.

#### miRNA and disease feature fusion

In the miRNA-disease feature fusion stage, the low-dimensional features of miRNA and disease from the previous stage were first obtained. Element-level addition, element-level multiplication, and concatenation were used to obtain the new miRNA-disease pair feature. Firstly, Vaswani et al. [40] proposed that positional coding and word embedding are commonly fused using additive fusion in Transformer, showing that additive fusion can effectively fuse information from different sources without destroying the structure. Moreover, Chen et al. [41] proposed a drug-target prediction model that uses additive fusion of drug and target features to simulate the ‘co-occurrence effect’ of the two. Therefore, we adopt the element-level addition method to achieve additive fusion of features by summing up the feature values of the corresponding dimensions, which helps to capture the linear complementary relationship between miRNAs and disease features. The calculation process is as follows:

20 $$\begin{aligned} {X_{add}} = {F_{miRNA}} + {F_{disease}} \end{aligned}$$

Second, He et al. [42] proposed that in neural collaborative filtering, user-item embedding vectors capture interaction features using element-level multiplication, which can enhance recommendation effectiveness. Therefore, we use element-level multiplication to capture nonlinear interactions by multiplying the corresponding dimensions to emphasise the dimensions that are simultaneously activated in the two feature vectors.

21 $$\begin{aligned} {X_{multi}} = {F_{miRNA}} \times {F_{disease}} \end{aligned}$$

Furthermore, Baltrušaitis et al. [43] verified in multimodal learning that splicing is a common underlying fusion strategy that preserves the complete information of each modality. The join operation simply splices the two feature vectors, preserving the respective original information and avoiding information loss in the fusion operation.

22 $$\begin{aligned} {X_{concat}} = Concat({F_{miRNA}},{F_{disease}}) \end{aligned}$$

Finally, we stitch these three fusion results into a comprehensive feature vector X so that the subsequent Transformer encoder can simultaneously utilise additive, multiplicative and raw feature information for correlation prediction, and the calculation process is as follows:

23 $$\begin{aligned} X = Concat({X_{add}},{X_{multi}},{X_{concat}}) \end{aligned}$$

#### miRNA and disease feature prediction

During the miRNA–disease association-prediction stage, the fused miRNA–disease-pair features are further refined by a standard Transformer encoder layer. This layer is composed of four tightly coupled sub-modules—multi-head self-attention (MHSA), layer normalization (LN), residual connection, and a position-wise feed-forward network (FFN)—stacked in a way that guarantees gradient stability while capturing long-range dependencies and fine-grained feature interactions. Specifically, to quantify the heterogeneous contribution of each feature dimension to the final prediction, the input representation is first fed into MHSA. By computing h parallel scaled dot-product attention heads, the model evaluates pairwise feature relevance in multiple complementary subspaces and produces a context-aware representation that is adaptively re-weighted. The subsequent LN and residual connection jointly normalize and add the attention output to the original input, effectively mitigating training degradation in deep architectures. Finally, the FFN distills higher-order non-linear patterns through two successive linear transformations separated by a non-linear activation, yielding a more discriminative embedding for downstream classification. The complete multi-head attention procedure is formally described as follows.

24 $$\begin{aligned} & {Q_i} = X \times {W_i}^Q \end{aligned}$$

25 $$\begin{aligned} & {K_i} = X \times {W_i}^K \end{aligned}$$

26 $$\begin{aligned} & {V_i} = X \times {W_i}^V \end{aligned}$$

27 $$\begin{aligned} & hea{d_i} = s\mathrm{{oftmax}}(\frac{{{Q_i} \times {K_i}^T}}{{\sqrt{{d_k}} }}) \end{aligned}$$

28 $$\begin{aligned} & {X_{mh}} = Concat(hea{d_1},hea{d_2},...,hea{d_n}){W^O} \end{aligned}$$

where ${Q_i}$, ${K_i}$, and ${V_i}$ are the matrices obtained from the linear transformation of the fused feature combination *X*, respectively. It is worth noting that the computation of Multi-source Feature Attention in the first stage is similar but differs slightly due to the different structures of the input features.

To counteract the intrinsic class-imbalance problem inherent in multi-class association prediction and to intensify the learning of information-rich yet hard-to-converge “difficult” samples, Focal Loss is employed as the dominant classification objective during training. By dynamically re-weighting easy versus hard instances, the loss steers the model toward iteratively focusing on ambiguous samples lying near the decision boundary, thereby markedly improving the recognition accuracy of minority-class associations. Moreover, since the dual-branch auto-encoders are responsible not only for feature extraction but also for dimensionality reduction and faithful reconstruction of the multi-source, high-dimensional miRNA and disease inputs, an auxiliary mean-squared-error (MSE) loss is additionally imposed to guarantee that the low-dimensional latent space preserves the original similarity structure. Consequently, the framework jointly optimises these two objectives, simultaneously boosting classification performance and representation quality; the overall composite loss is formally defined as follows.

29 $$\begin{aligned} & {L_{cls}} = - ({y_{ij}}(1 - {\hat{y}_{ij}})log({\hat{y}_{ij}}) + (1 - {y_{ij}}){\hat{y}_{ij}}log(1 - {\hat{y}_{ij}})) \end{aligned}$$

30 $$\begin{aligned} & {L_{auxiliary1}} = {\left\| {({o_{mfs}},{o_{mgs}},{o_{mis}}) - ({x_{MFS}},{x_{MGS}},{x_{MIS}})} \right\| ^2} \end{aligned}$$

31 $$\begin{aligned} & {L_{auxiliary2}} = {\left\| {({o_{dss}},{o_{dgs}},{o_{dis}}) - ({x_{DSS}},{x_{DGS}},{x_{DIS}})} \right\| ^2} \end{aligned}$$

32 $$\begin{aligned} & L = {L_{cls}} + {L_{auxiliary1}} + {L_{auxiliary2}} \end{aligned}$$

where $y_{ij}$ represents the true label associated with $m_i$ and $d_j$, while ${\hat{y}_{ij}}$ represents the prediction score of our method for it.

## Results

### Cross validation

On the HMDD v2.0 dataset, we conducted 5-fold cross-validation (5-CV) and 10-fold cross-validation (10-CV) experiments tocomprehensively evaluate the performance of MFCAMNet. Additionally, 5-fold cross-validation experiments are conducted on the HMDD v3.2 dataset to assure the model generalization ability. In the 5-fold cross-validation, all known miRNA-disease associations will be considered positive samples and randomly divided into five groups, with each group serving as a test set in turn and the remaining four groups serving as a train set. 10-fold cross-validation is similar to the above and our method also generates the same number of negative miRNA-disease samples from unknown associations as positive samples. For comparison with other methods, we measured performance primarily with six commonly employed evaluation metrics: area under the receiver operating characteristic (ROC) curve (AUC), area under the precision/recall (PR) curve (AUPR), accuracy, precision, recall, and F1 score. AUC describes the possibility that the model ranks positive samples higher than negative samples, whereas AUPR describes the relationship between precision and recall at different thresholds. AUC and AUPR are usually the two most useful metrics for evaluating the performance of a model. Greater AUC and AUPR indicate better model performance. The remaining four evaluation metrics are defined as follows:

33 $$\begin{aligned} & Accuracy = \frac{{TP + TN}}{{TP + TN + FP + FN}} \end{aligned}$$

34 $$\begin{aligned} & \mathrm{{Precision}} = \frac{{TP}}{{TP + FP}} \end{aligned}$$

35 $$\begin{aligned} & \mathrm{{Recall}} = \frac{{TP}}{{TP + FN}} \end{aligned}$$

36 $$\begin{aligned} & F1 = 2 \times \frac{{\mathrm{{Precision}} \times \mathrm{{Recall}}}}{{\mathrm{{Precision + Recall}}}} \end{aligned}$$

where *TP* is the number of correctly identified positive samples, and *FN* is the number of incorrectly identified positive samples. *FP* represents the number of falsely identified negative samples, and *TN* represents the number of correctly identified negative samples.

### Performance comparison with other methods

Currently, many computational methods have been developed for miRNA-disease association prediction. To ensure the comprehensiveness and fairness of the evaluation, we selected the comparison experiments based on algorithmic diversity, reproducibility and data consistency. In this paper, we compare MFCAMNet with 12 other methods, namely VAEMDA [17], GBDT-LR [21], ABMDA [22], AEMDA [30], SAEMDA [31], MINIMDA [33], EDTMDA [44], NIMCGCN [45], ERMDA [46], GAEMDA [47], VGAEMDA [48], PATMDA [49] and EMCMDA [50]. For a fair comparison, we reimplemented all comparison methods and evaluated them using the same training/testing division as in this experiment.

**Fig. 3:**
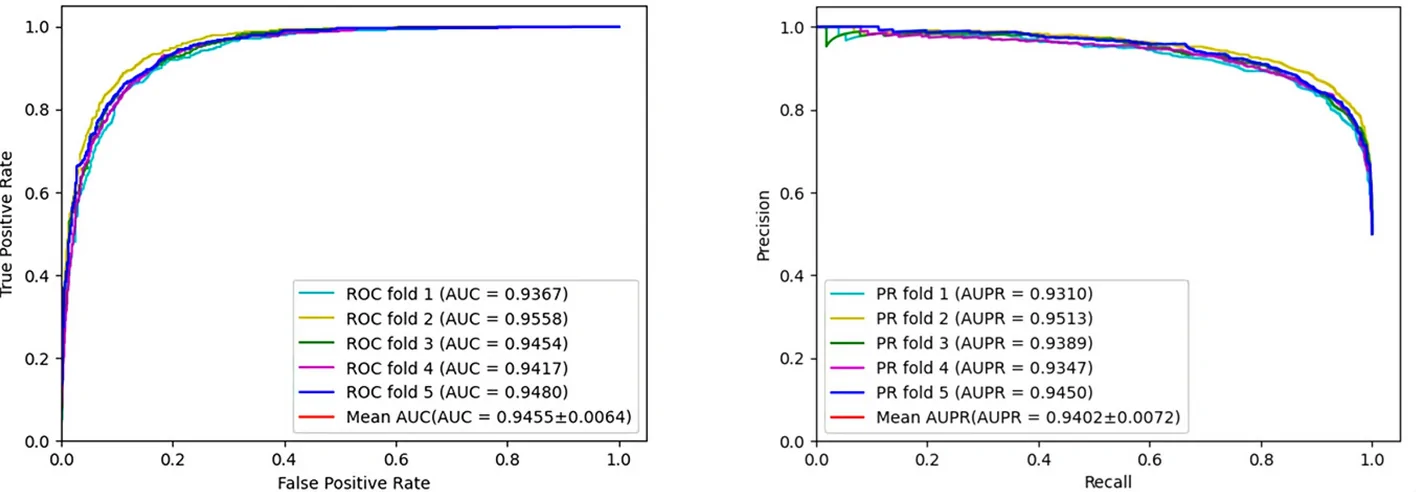
ROC curve and PR curve performed by MFCAMNet under 5-CV on HMDD v2.0 dataset

**Fig. 4:**
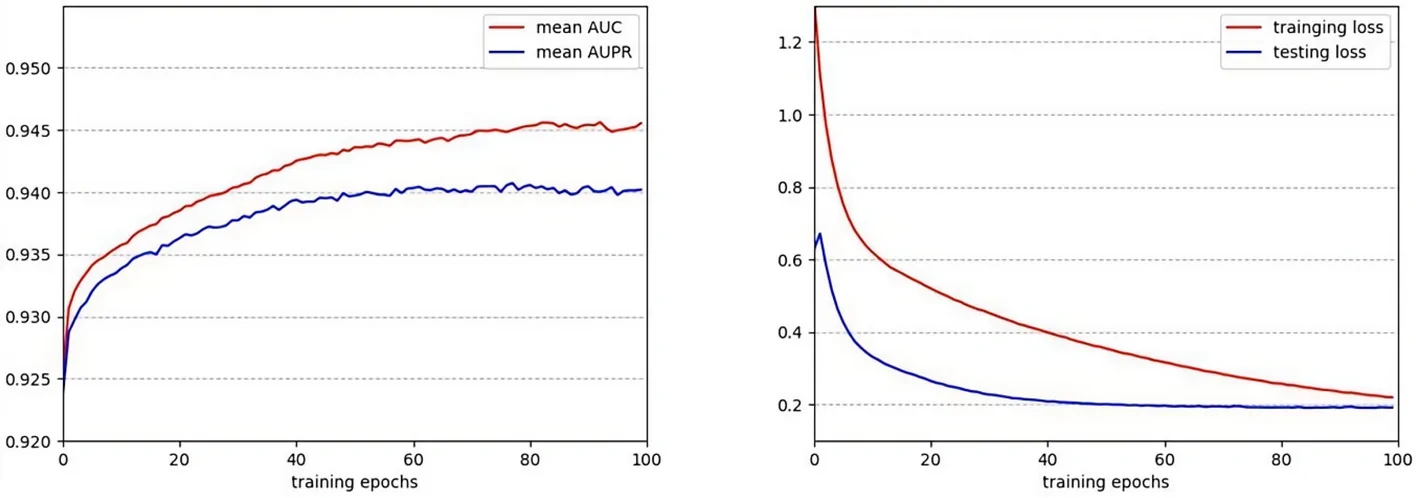
MFCAMNet was trained on HMDD2.0 using 5-cv for 100 epochs. The results show gradual improvement between AUC and AUPR (left) and a gradual decrease in loss (right)

- VAEMDA [17] trained the variational autoencoder (VAE) based on two spliced matrices and obtained final predicted association scores between miRNAs and diseases were obtained by integrating the scores from the two trained VAE models.
- GBDT-LR [21] adopted the gradient boosting decision tree (GBDT) model to obtain effective embeddings and predict the final miRNA-disease association score by the logistic regression (LR) model.
- ABMDA [22] generated balanced positive and negative samples and used a decision tree classifier to infer the association between miRNAs and diseases.
- AEMDA [30] first extracted the dense and highdimensional features of miRNAs and diseases and employed the deep autoencoder without negative samples to retrieve the underlying associations between miRNA and disease.
- SAEMDA [31] pre-trained stacked autoencoders (SAEMDA) unsupervised using all known and unknown miRNA pairs. Then, fine-tune SAEMDA in supervised methods to predict latent association.
- MINIMDA [33] developed a new computational method that utilizes multiple sources of information to construct multiple information networks and then obtains feature representations of miRNA and diseases by mixing neighborhood information from the multiple networks.
- EDTMDA [44] comprehensively learned the embedding of miRNA–disease pair by statistical measures, graph theoretical measures and matrix factorization results.
- NIMCGCN [45] employed a graph convolutional network (GCN) to learn latent feature representations of miRNAs and diseases from two similarity networks and then applied a neural inductive matrix completion (NIMC) models to predict their latent association.
- ERMDA [46] was the ensemble learning method to learn the feature representation of miRNAs and disease by integrating similarity from multiple balanced training subsets and using multiple independent learners to predict miRNA-disease associations jointly.
- GAEMDA [47] employed a GCN to learn the low lowdimensional embeddings of miRNA and disease by aggregating neighborhood information. Then, the embeddings of miRNA and disease nodes are fed into a bilinear decoder to identify the potential links between miRNA and disease.
- VGAEMDA [48] first obtained miRNA and disease features from heterogeneous networks and then two variational graph autoencoders (VGAE) were used to calculate miRNA-disease association scores.
- PATMDA [49] designed a computational method that performed random walks with restart and point-wise mutual information on multiple similarity networks and used convolutional neural networks to obtain features of miRNAs and diseases.
- EMCMDA [50] is a matrix completion method based on minimizing the truncated Schatten p-norm. It constructs heterogeneous networks by integrating multi-source similarity and employs a weighted singular value reduction algorithm to dynamically adjust singular values to preserve physical meaning, thereby enabling the prediction of miRNA-disease associations.
- DMHLF [51] achieves efficient prediction of disease-related ceRNA biomarkers through a dual-channel mechanism combining feature-driven hypergraph-weighted dynamic random walks and structure-driven Eigen-GNN spectral analysis.

**Fig. 5:**
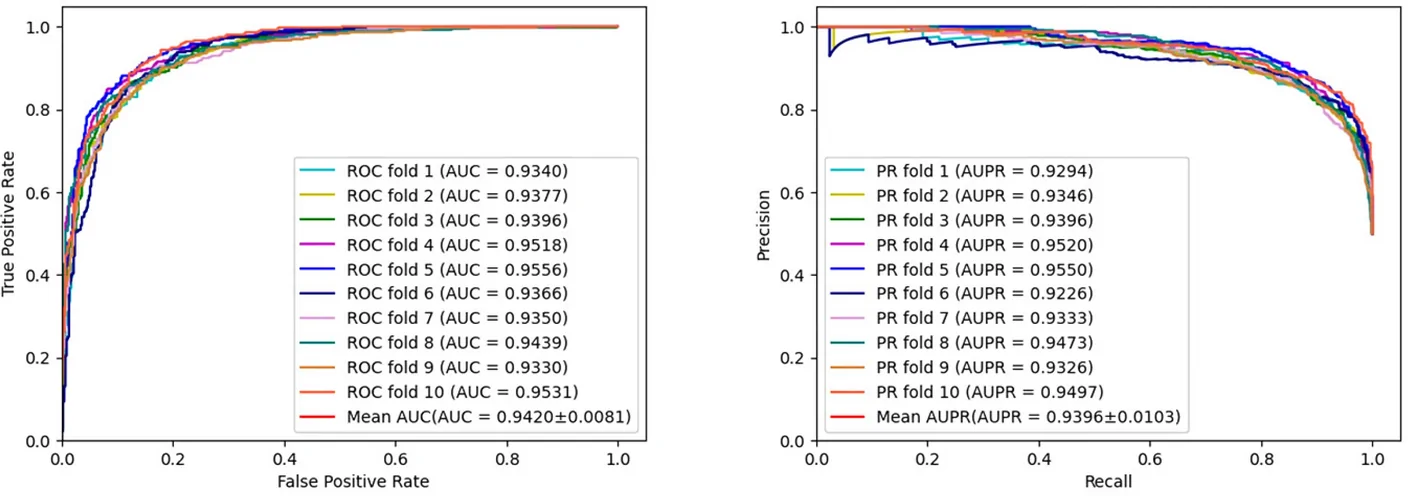
ROC curve and PR curve performed by MFCAMNet under 10-CV on HMDD v2.0 dataset

**Fig. 6:**
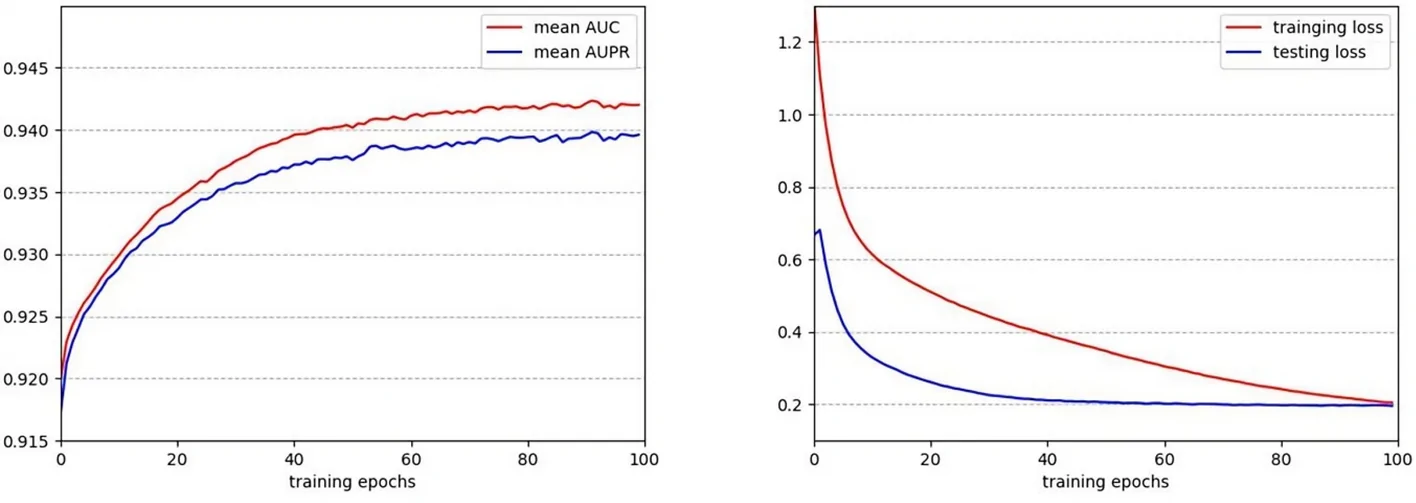
MFCAMNet was trained on HMDD2.0 using 10-cv for 100 epochs. The results show gradual improvement between AUC and AUPR (left) and a gradual decrease in loss (right)

As shown in Fig. 3 and Table 2, MFCAMNet outperformed the other methods in terms of AUC, AUPR and Precision metrics. The proposed model yields mean AUC of 0.9455, mean AUPR of 0.9402 and Precision of 0.8655. It obtained 0.8715 and 0.8727 for the Accuracy and F1 metrics to place 2nd behind MINIMDA, respectively, but 0.8801 for the Recall measure ranked 9th overall. Furthermore, Fig. 4 depicts the performance of model changes during training. the Training process which shows that the model gradually converges after 40 training epochs and the training and test loss follow the same trend. The results of the 10-CV experiment are displayed in Fig. 5, where MFCAMNet achieves a mean AUC of 0.9420±0.0081 and a mean AUPR of 0.9396±0.0103. These results demonstrate that the prediction capacity on the HMDD v2.0 dataset is both effective and accurate.

**Table 2:** Results of prediction performance for each method under 5-CV on HMDD v2.0 dataset

Method	AUC	AUPR	Accuracy	Precision	Recall	F1
AEMDA	0.9134	0.9127	0.8371	0.8102	0.8805	0.8438
ABMDA	0.8760	0.8620	0.8069	0.7669	0.8848	0.8208
GBDT-LR	0.8618	0.8556	0.8015	0.7690	0.8634	0.8130
EDTMDA	0.9137	0.9160	0.8423	0.8353	0.8535	0.8440
VAEMDA	0.9260	0.9215	0.8382	0.8045	0.8938	0.8466
NIMCGCN	0.9172	0.9186	0.8438	0.8162	0.8886	0.8504
ERMDA	0.9013	0.9043	0.8330	0.8096	0.8639	0.8356
GAEMDA	0.9307	0.9267	0.8522	0.8229	0.8988	0.8588
SAEMDA	0.9154	0.9116	0.8391	0.8065	0.8923	0.8472
VGAEMDA	0.9385	0.9363	0.8713	0.8445	0.9103	0.8706
MINIMDA	0.9444	0.9296	0.8789	0.8545	0.9160	0.8839
PATMDA	0.9330	0.9340	0.8575	0.8527	0.8653	0.8588
EMCMDA	0.9352	0.9328	0.8658	0.8386	0.9056	0.8708
DMHLF	0.9448	0.9386	0.8704	0.8523	0.9138	0.8822
MFCAMNet	0.9455	0.9402	0.8715	0.8655	0.8801	0.8727

**Fig. 7:**
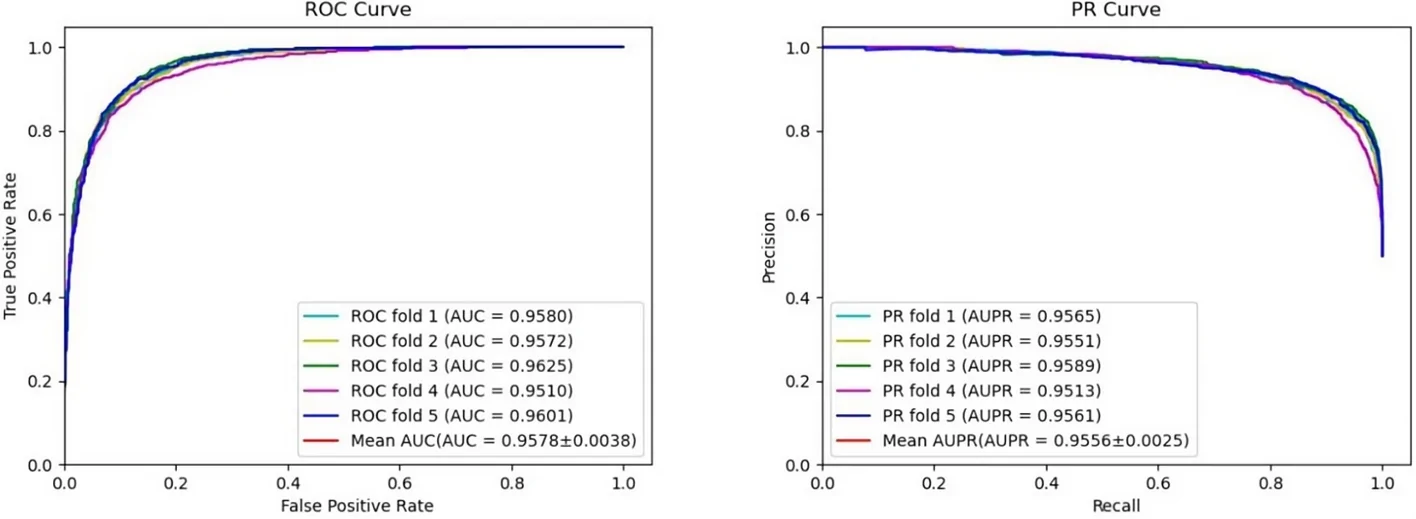
ROC curve and PR curve performed by MFCAMNet under 5-CV on HMDD v3.2 dataset

**Fig. 8:**
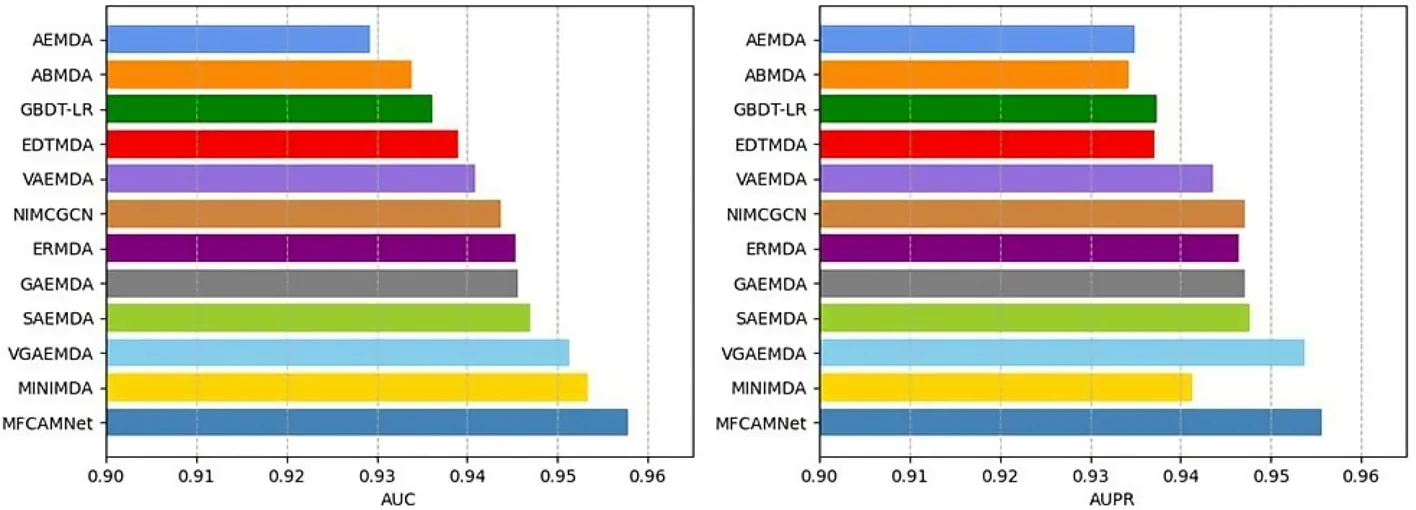
Result of prediction performance for each method under 5-CV on HMDD v3.2 dataset

**Fig. 9:**
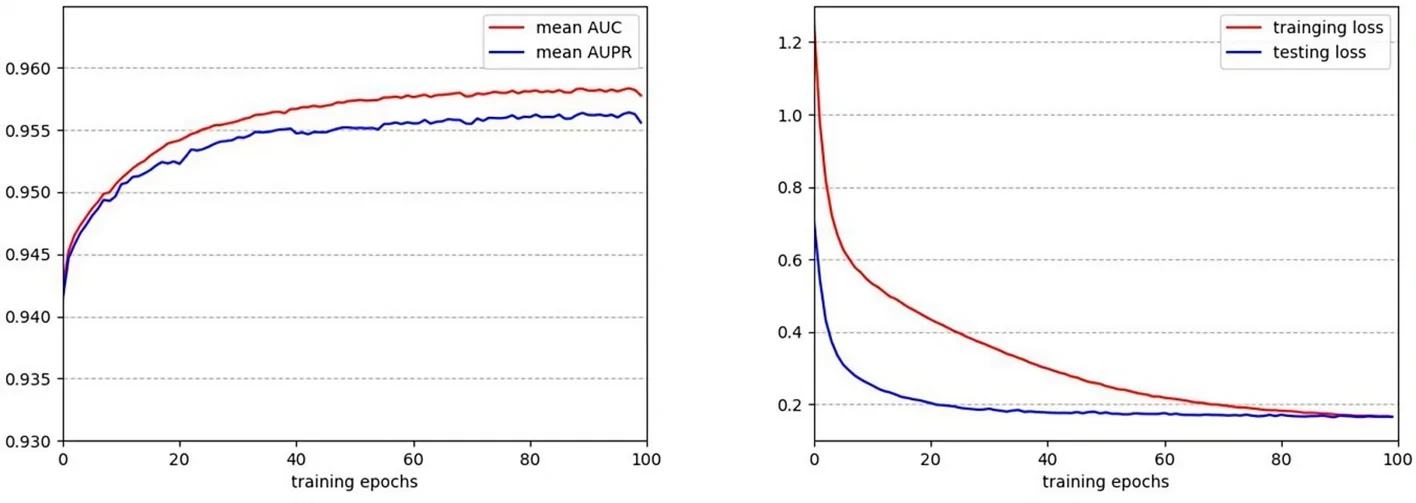
MFCAMNet was trained on HMDD3.2 using 5-cv for 100 epochs. The results show gradual improvement between AUC and AUPR (left) and a gradual decrease in loss (right)

As shown in Table 3, MFCAMNet outperformed the other methods in terms of AUC, AUPR, and Precision metrics. It ranked 3rd behind MINIMDA and VGAEMDA with scores of 0.8685 and 0.8700 for Accuracy and F1, but only 8th with 0.8803 for Recall. Similarly, we examined the changes in our proposed model’s performance metrics during training. As the number of training epochs rises, as shown in Fig. 6, our model continues to improve in terms of the performance metrics AUC and AUPR, and we can also observe that the training loss and test loss are both steadily declining and converging from the peak. These are positive signs that our model is stable and performing well. Besides, we tested the performance of the model on other datasets to ensure its reliability and accuracy, gain insight into the strengths and shortcomings of the model, and make the required improvements to improve its performance. As demonstrated in Fig. 7, we conducted a 5- fold cross-validation experiment on the HMDD v3.2 dataset, and the proposed model performed well, with a mean AUC of 0.9578±0.0038 and an AUPR of 0.9556±0.0025. When compared to other methods, the results presented in Fig. 8 reveal that our MFCAMNet model batter the other methods and obtained the best outcomes in both the AUC and AUPR metrics. Figure 9 depicts the training process of our proposed model on the HMDD3.2 dataset using a 5-fold cross-validation experiment, with results similar to the above experiments. The model rapidly increases in the AUC and AUPR metrics over the first 40 training epochs. The model began to converge slowly and progressively after 40 training epochs. Overall, all results demonstrated that our method is capable of making precise predictions about potential miRNA-disease relationships among various datasets.

Through the observation of Tables 2 and 3, the performance of MFCAMNet and MINIMDA is close to each other in terms of the core indexes, which is essentially that both of them have captured the core value of multi-source fusion in the miRNA-disease association prediction task, a phenomenon that confirms that making full use of the multi-dimensional features is the key to improve the prediction performance. The unique advantage of MFCAMNet lies in its innovative cascade attention, triple feature fusion and double loss function design. Compared with MINIMDA, which focuses on the neighbourhood aggregation of network topology, its innovative cascade attention, triple feature fusion and double loss function design focuses on the refinement of heterogeneous data, which is the core reason for its more significant performance on the larger HMDD v3.2 dataset. on larger HMDD v3.2 datasets.

**Table 3:** Results of prediction performance for each method under 10-CV on HMDD v2.0 dataset

Method	AUC	AUPR	Accuracy	Precision	Recall	F1
AEMDA	0.9159	0.9169	0.8411	0.8213	0.8725	0.8454
ABMDA	0.8811	0.8725	0.8115	0.7732	0.8847	0.8243
GBDT-LR	0.8669	0.8645	0.8077	0.7809	0.8573	0.8618
EDTMDA	0.9193	0.9198	0.8416	0.8138	0.8878	0.8485
VAEMDA	0.9215	0.9204	0.8496	0.8237	0.8908	0.8554
NIMCGCN	0.9188	0.9192	0.8494	0.8331	0.8763	0.8523
ERMDA	0.9052	0.9088	0.8390	0.8528	0.8605	0.8424
GAEMDA	0.9328	0.9280	0.8587	0.8303	0.9022	0.8644
SAEMDA	0.9157	0.9122	0.8399	0.8143	0.8816	0.8461
VGAEMDA	0.9388	0.9360	0.8718	0.8511	0.9012	0.8706
MINIMDA	0.9406	0.9276	0.8746	0.8408	0.9246	0.8804
PATMDA	0.9184	0.9210	0.8528	0.8426	0.8541	0.8422
EMCMDA	0.9336	0.9305	0.8623	0.8342	0.9013	0.8664
DMHLF	0.9412	0.9372	0.8629	0.8476	0.9095	0.8778
MFCAMNet	0.9420	0.9396	0.8685	0.8605	0.8803	0.8700

### Paraments analysis

**Fig. 10:**
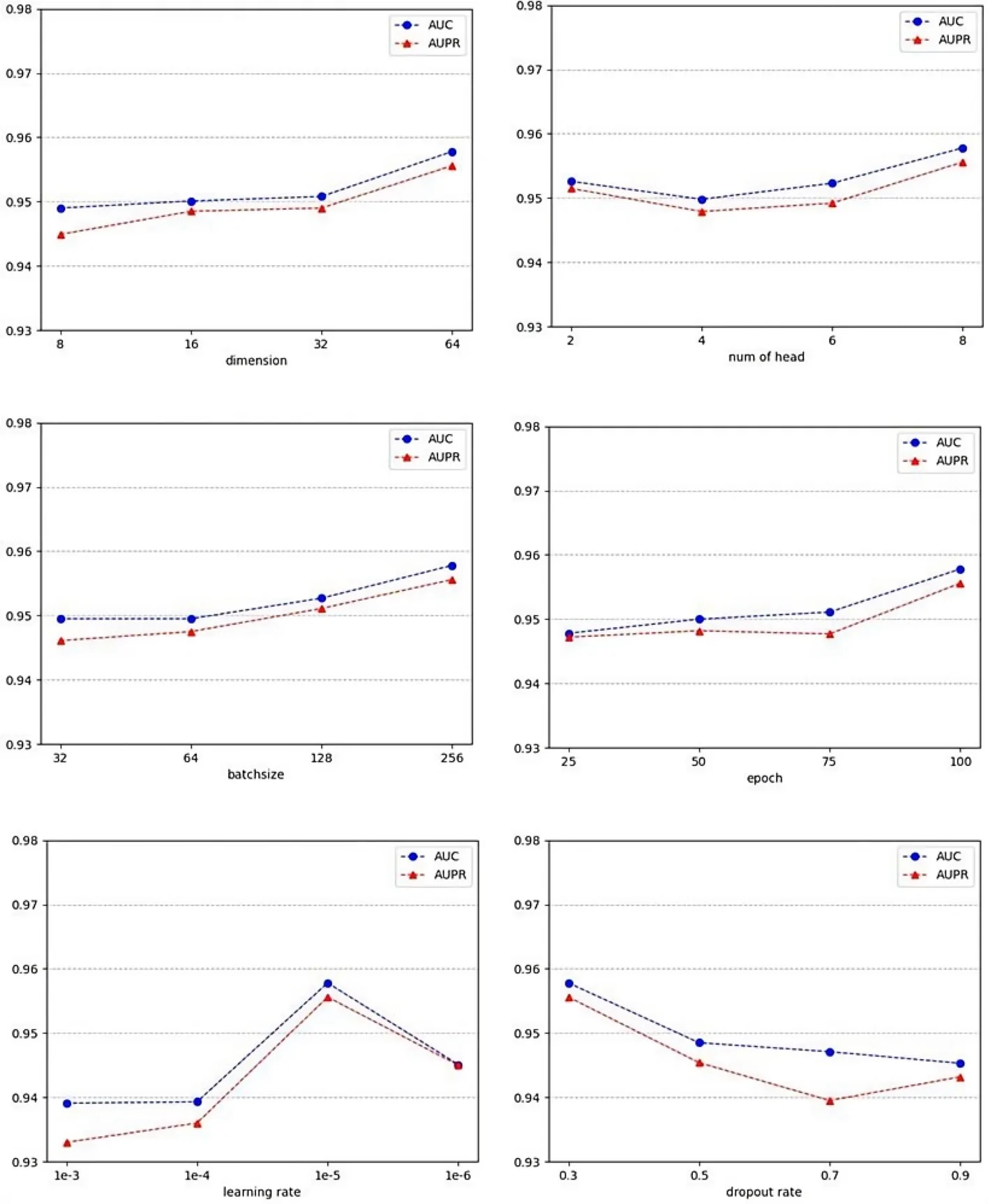
The results of MFCAMNet on the AUC and AUPR metrics with different hyperparameters on the HMDD v3.2 dataset

Hyper-parameters in deep learning decisively influence predictive performance. To systematically assess their impact on our proposed model, we conducted five-fold cross-validation experiments on the HMDD v3.2 dataset, using the area under the ROC curve (AUC) and the area under the precision–recall curve (AUPR) as primary evaluation metrics. The hyper-parameters examined include the auto-encoder output dimension, the number of heads in multi-head attention, dropout rate, training epochs, batch size, and learning rate. As shown in Fig. 10, the model is most sensitive to changes in learning rate and dropout, while adjustments to the remaining parameters yield only gradual improvements in AUC and AUPR. Guided by these observations, the optimal configuration was determined as follows: auto-encoder output dimension 64, multi-head attention heads 8, batch size 256, training epochs 100, dropout 0.3, and learning rate 1e-5.

**Fig. 11:**
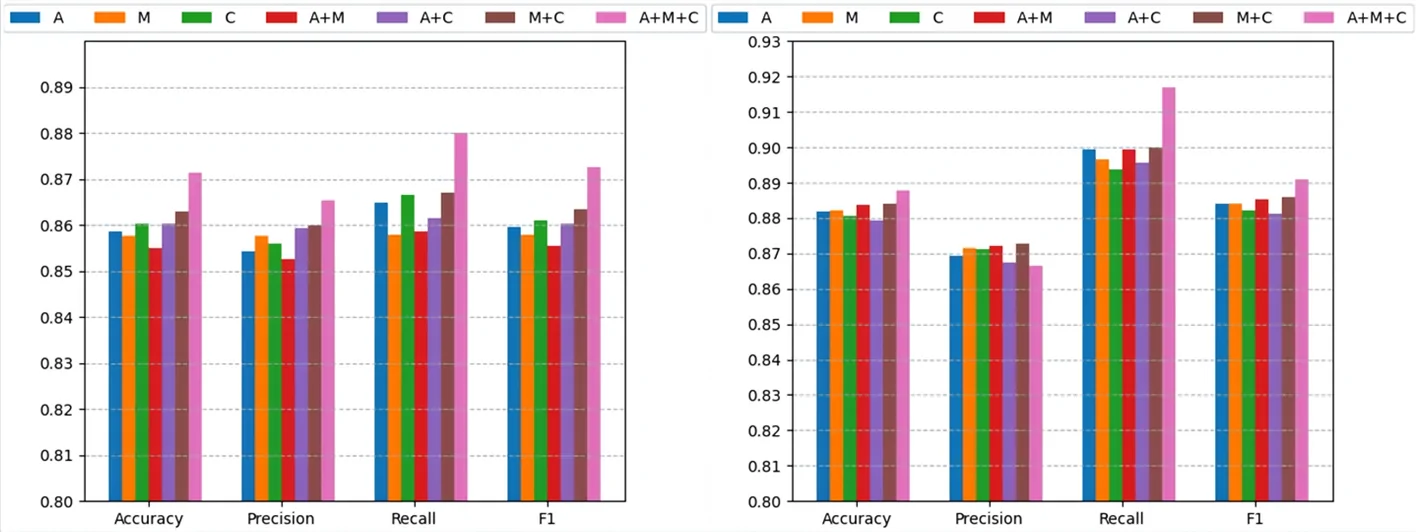
Performance result of MFCAMNet that use different ways to fuse miRNA and disease features. The left subfigure shows the results of the HMDD v2.0 dataset and the right subfigure shows the results of the HMDD v3.2 dataset. Where A represents element-level addition, M represents element-level multiplication, C represents concatenation and X+Y represents the combination of different fusion methods

### Comparison of different feature fusion

In our experiments, we used multiple source features, including miRNA functional similarity, disease semantic similarity, miRNA GIP similarity, disease GIP similarity, integrated similarity for miRNA and integrated similarity for disease. In order to explore the most appropriate feature fusion approach, we attempted to fuse multiple source features using different methods. Thus, seven MFCAMNet models were constructed to explore and compare the performance differences between the different fusion methods. The experimental results are shown in Fig. 11. In previous studies, the feature fusion of miRNAs and diseases frequently used only one of addition, multiplication, or concatenation. In this experiment, we combined miRNA and disease features in three ways to obtain the best results. On the HMDD v2.0 dataset, as shown in Fig. 11, the combination of addition, multiplication, and concatenation yields the best results in the four performance metrics. The second-best results are obtained by combining multiplication and concatenation. Similarly, the experimental results on the HMDD v3.2 dataset show that the combination of addition, multiplication, and concatenation produces the best results in the accuracy, recall, and F1 metrics, with multiplication and concatenation producing the second-best results. Based on the results of the above experiments, the combination of addition, multiplication and concatenation seems to be the best strategy for miRNA-disease feature fusion and retains the most information. This also shows that the use of multi-source features and the fusion of multi-source features may provide deep learning models with diverse information from different perspectives, thereby improving the accuracy of miRNA-disease association prediction compared to previous work using single-feature information.

### Ablation study

**Fig. 12:**
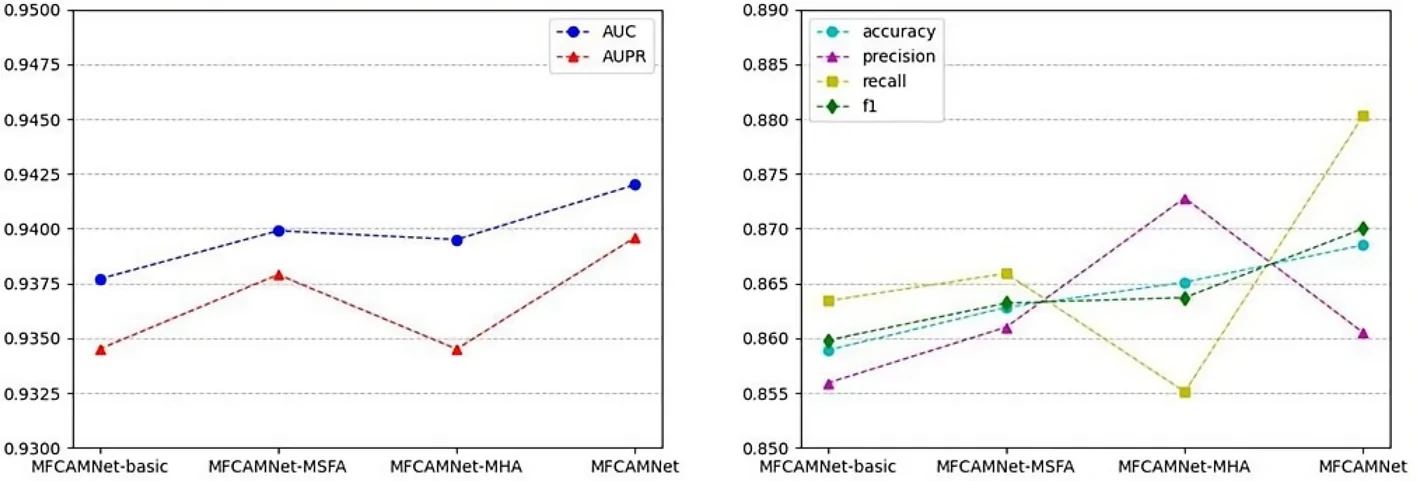
Comparison of the results of MFCAMNet and its variant models on the HMDD2.0 dataset using 10-fold CV

To rigorously quantify the contribution of the attention modules within MFCAMNet, we devised and compared three architectural variants: MFCAMNet-basic, MFCAMNet-MSFA, and MFCAMNet-MHA. MFCAMNet-basic completely removes all attention components; MFCAMNet-MSFA retains only the Multisource Feature Attention (MSFA) module in the miRNA and disease representation learning stage; MFCAMNet-MHA introduces Multi-Head Attention (MHA) exclusively in the association prediction stage via encoder layers, enabling the model to identify salient predictive signals by learning the weight distribution over miRNA–disease pair features. As illustrated in Fig. 12, the incorporation of attention mechanisms yields consistent and substantial improvements across all evaluation metrics, corroborating the indispensability and efficacy of the attention modules in enhancing the discriminative capacity of MFCAMNet.

### Case study

To evaluate the potential of MFCAMNet to predict novel miRNA-disease associations, we chose three diseases as case studies: breast cancer, esophageal cancer and lung cancer. Understanding the role of miRNAs in various diseases may lead to advances in diagnosis and therapy. In our experiments, MFCAMNet was trained with 5430 known miRNA-disease associations from HMDD v2.0. The trained model was then used to calculate the association scores of candidate miRNAs with specific diseases. The predicted association scores were ranked in descending order. The top 30 results were filtered and then validated using two additional authoritative datasets: HMDD v3.2 and dbDEMC v3.0. The latter is a comprehensive database of human diseases and their associated miRNAs, including 3268 differentially expressed miRNAs from 40 different cancer types.

**Table 4:** Case study for esophageal cancer

Rank	MiRNA	Score	Evidence	Rank	MiRNA	Score	Evidence
1	hsa-mir-17	0.9695	I,II	16	hsa-let-7i	0.9522	I,II
2	hsa-mir-125b	0.9674	I,II	17	hsa-mir-122	0.9521	I,II
3	hsa-mir-221	0.9650	I,II	18	hsa-mir-1	0.9515	I,II
4	hsa-mir-18a	0.9642	I,II	19	hsa-mir-181b	0.9507	I,II
5	hsa-mir-19b	0.9629	II	20	hsa-mir-133b	0.9502	I,II
6	hsa-mir-222	0.9628	I,II	21	hsa-mir-181a	0.9501	II
7	hsa-mir-142	0.9559	I,II	22	hsa-let-7d	0.9498	II
8	hsa-mir-200b	0.9541	I,II	23	hsa-mir-9	0.9522	I,II
9	hsa-mir-29a	0.9530	II	24	hsa-mir-7	0.9521	I,II
10	hsa-mir-29b	0.9522	II	25	hsa-let-7f	0.9515	II
11	hsa-let-7e	0.9507	I,II	26	hsa-mir-124	0.9507	I,II
12	hsa-mir-106b	0.9502	I,II	27	hsa-let-7g	0.9502	I,II
13	hsa-mir-16	0.9498	I,II	28	hsa-mir-10b	0.9501	I,II
14	hsa-mir-146b	0.9483	I,II	29	hsa-mir-182	0.9498	II
15	hsa-mir-93	0.9483	I,II	30	hsa-mir-106a	0.9522	I,II

**Table 5:** Case study for breast cancer

Rank	MiRNA	Score	Evidence	Rank	MiRNA	Score	Evidence
1	hsa-mir-142	0.9763	I,II	16	hsa-mir-92b	0.9583	I,II
2	hsa-mir-150	0.9730	I,II	17	hsa-mir-99b	0.9580	II
3	hsa-mir-106a	0.9728	I,II	18	hsa-mir-372	0.9545	I,II
4	hsa-mir-15b	0.9722	I,II	19	hsa-mir-32	0.9518	I,II
5	hsa-mir-138	0.9711	I,II	20	hsa-mir-130b	0.9508	I,II
6	hsa-mir-99a	0.9693	I,II	21	hsa-mir-370	0.9495	I,II
7	hsa-mir-378a	0.9688	I,II	22	hsa-mir-212	0.9493	I,II
8	hsa-mir-192	0.9684	I,II	23	hsa-mir-424	0.9441	I,II
9	hsa-mir-196b	0.9669	I,II	24	hsa-mir-330	0.9436	I,II
10	hsa-mir-98	0.9638	I,II	25	hsa-mir-186	0.9431	II
11	hsa-mir-130a	0.9636	I,II	26	hsa-mir-144	0.9407	I,II
12	hsa-mir-181c	0.9632	I,II	27	hsa-mir-211	0.9398	I,II
13	hsa-mir-30e	0.9628	I,II	28	hsa-mir-208a	0.9384	I,II
14	hsa-mir-574	0.9591	I,II	29	hsa-mir-28	0.9383	II
15	hsa-mir-181d	0.9590	II	30	hsa-mir-449a	0.9378	I,II

Esophageal cancer is the eighth most commonly diagnosed cancer and the sixth most common cause of cancer death worldwide, with high morbidity and mortality rates and an overall poor prognosis. The incidence and mortality of esophageal cancer has increased dramatically. By 2020, there will be over 600,000 new cases and over 540,000 deaths worldwide [52]. Early detection and treatment may help to improve patient survival. It has been shown that miRNA-19b is positively correlated with tumor size, miRNA-17a overexpression is positively correlated with lymph node metastasis, and miRNA-18a expression is also correlated with tumor stage [8]. Based on these findings, we calculated the association scores of all potential miRNAs with esophageal cancer and Table 4 shows the top 30 predicted association scores, verified by HMDD v3.2 (I) or dbDEMC3.0 (II).

Breast cancer is a serious health problem for women worldwide, with over 2.26 million new cases and 685,000 case deaths predicted in 2020 [53]. The morbidity and mortality rates of breast cancer are strongly correlated with the level of local development. Morbidity rates are significantly higher in high development countries than in low development countries, while overall 5-year survival rates are much higher in high development countries due to early detection, early diagnosis, and more effective treatment. Thus, although the morbidity of breast cancer is rapidly increasing worldwide, its higher mortality rate is not inevitable. Previous studies have shown that breast cancer is strongly associated with several miRNAs, such as miRNA-195 and miRNA-497, which are negatively correlated with the malignancy of breast cancer. Table 5 demonstrates the predicted results of the association of candidate miRNAs with breast cancer, with the majority of the results validated by HMDD3.2 (I) and dbDEMC3.0 (II) can validate all predictions.

The third disease in the case study is lung cancer. Lung cancer is the most commonly diagnosed cancer worldwide and the leading cause of cancer-related death [54]. The majority of patients are considered to be associated with smoking. By examining the role of miRNAs in lung cancer development, it was found that some miRNA expression is strongly associated with lung carcinogenesis. For example, miRNA- 17-92 cluster expression levels were significantly increased in lung cancer cells. miRNA-155 abnormal expression was commonly found in various cancers, including lung cancer, and miRNA-29 downregulation may contribute to lung cancer development. MFCAMNet used known miRNA-disease association informa-tion to predict the probability of miRNAs with lung cancer, and used HMDD3.2 and deDEMC3.0 were used to validate. The results are shown in Table 6. 28 of the first 30 results can be validated by HMDD3.2 (I), and dbDEMC3.0 (II) can validate all the results.

**Table 6:** Case study for lung cancer

Rank	MiRNA	Score	Evidence	Rank	MiRNA	Score	Evidence
1	hsa-mir-17	0.9695	I,II	16	hsa-let-7i	0.9522	I,II
2	hsa-mir-125b	0.9674	I,II	17	hsa-mir-122	0.9521	I,II
3	hsa-mir-221	0.9650	I,II	18	hsa-mir-1	0.9515	I,II
4	hsa-mir-18a	0.9642	I,II	19	hsa-mir-181b	0.9507	I,II
5	hsa-mir-19b	0.9629	II	20	hsa-mir-133b	0.9502	I,II
6	hsa-mir-222	0.9628	I,II	21	hsa-mir-181a	0.9501	II
7	hsa-mir-142	0.9559	I,II	22	hsa-let-7d	0.9498	II
8	hsa-mir-200b	0.9541	I,II	23	hsa-mir-9	0.9522	I,II
9	hsa-mir-29a	0.9530	II	24	hsa-mir-7	0.9521	I,II
10	hsa-mir-29b	0.9522	II	25	hsa-let-7f	0.9515	II
11	hsa-let-7e	0.9507	I,II	26	hsa-mir-124	0.9507	I,II
12	hsa-mir-106b	0.9502	I,II	27	hsa-let-7g	0.9502	I,II
13	hsa-mir-16	0.9498	I,II	28	hsa-mir-10b	0.9501	I,II
14	hsa-mir-146b	0.9483	I,II	29	hsa-mir-182	0.9498	II
15	hsa-mir-93	0.9483	I,II	30	hsa-mir-106a	0.9522	I,II

As shown in Table 7, among breast cancer patients, the high-expression groups of miR-150 and miR-99a exhibited a longer median survival time (142.2 months) compared to their low-expression counterparts (113.6 and 115.4 months, respectively). Similarly, high expression of miR-15b was associated with prolonged survival (142.2 vs. 131.5 months).

**Table 7:** Median survival time of top 6 predicted miRNA with different expression level in breast cancer

	hsa-mir-142	hsa-mir-150	hsa-mir-106a	hsa-mir-15b	hsa-mir-138	hsa-mir-99a
Low	129.1	131.5	124.53	131.5	215.2	115.4
High	113.63	142.23	131.37	142.23	113.63	142.23

In contrast, high expression of miR-138 significantly shortened survival (115.6 vs. 215.2 months), while the difference in survival between high and low expression of miR-142 and miR-106a was minimal. These findings suggest that miR-150, miR-99a, and miR-15b may serve as favorable prognostic factors, whereas miR-138 appears to be an unfavorable factor.

### Survival analysis

**Fig. 13:**
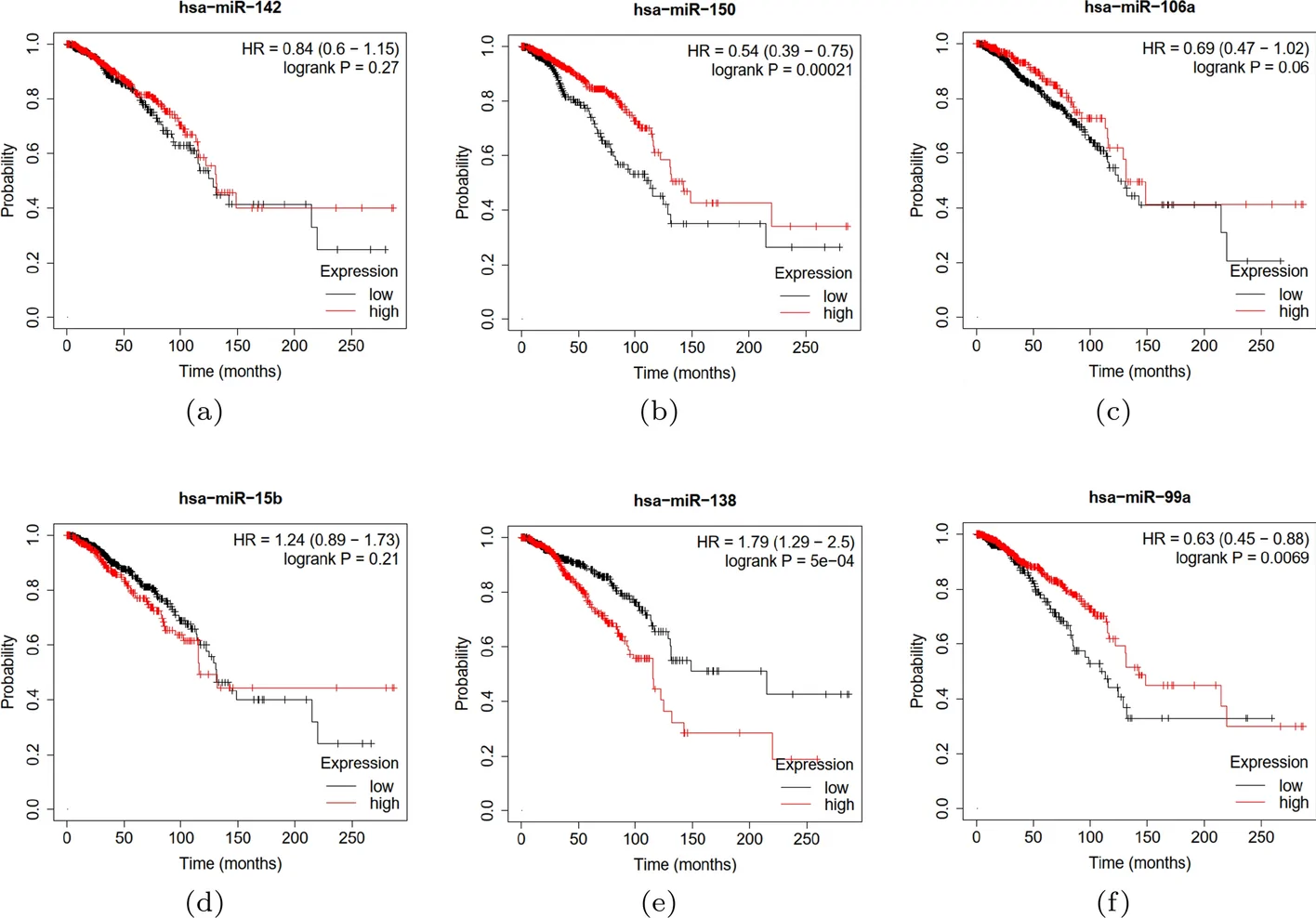
Survival analysis of top 6 predicted miRNA for breast cancer

We obtain clinical data related to breast cancer from the Cancer Genome Atlas database and conduct Kaplan-Meier survival analysis to validate whether the top 6 predicted miRNAs can be confirmed by the clinical data. The top 6 miRNAs are miRNA-142, miRNA-150, miRNA-106a, miRNA-15b, miRNA-138, and miRNA-99a, respectively. Survival analysis is help to identify whether these miRNAs have prognostic value in breast cancer patients.

The results are displayed in Fig. 13a–f. Breast cancer patients with higher miRNA-142 expression levels exhibited a slightly higher overall survival rate compared to patients with lower expression levels. The expression level of miRNA-150 is positively correlated with the overall survival rate of patients, with a median survival period of 142.23 months for breast cancer patients with high expression levels, while patients with low expression levels had a median survival period of only 113.63 months, representing a difference of 28.6 months. Research indicates that the overexpression of miRNA-150 affects genes related to tumorigenesis , yet it does not increase the generation, migration, or invasion of breast cancer cells. Instead, this overexpression is directly associated with better patient prognosis. These findings suggest that miRNA-150 may have potential therapeutic and prognostic value in breast cancer patients. This discovery provides important insights for a deeper understanding of the molecular mechanisms of breast cancer and the development of relevant treatment strategies, holding significant implications for improving the treatment and survival rates of breast cancer patients.

The overexpression of miRNA-106a is positively correlated with the overall survival rate of patients. In contrast, the overexpression of miRNA-15b shows a negative correlation with the overall survival rate. The median survival period of breast cancer patients with miRNA-15b overexpression is only 115.73 months, while those with low expression levels have an extended median survival period of 131.37 months. Studies suggest that miRNA-15b is often significantly over expressed in breast cancer patients, while the expression level of PAQR3 is relatively low. However, PAQR3 has been demonstrated to have anti-cancer effects in various tumors. Therefore, miRNA-15b may be involved in the pathological process of breast cancer by suppressing PAQR3 expression, thereby promoting the deterioration of breast cancer. Further research may contribute to the development of treatment strategies targeting miRNA-15b and PAQR3 to slow down or inhibit the progression of breast cancer, providing more effective treatment options for breast cancer patients.

The overexpression of miRNA-138 leads to a sharp decrease in the overall survival rate of breast cancer patients, with a median survival period of 215.2 months for patients with low expression levels and only 115.4 months for those with overexpression, a difference of nearly two-fold. In contrast, breast cancer patients with overexpression of miRNA- 99a exhibit higher overall survival rates compared to patients with low miRNA-99a expression levels. Studies indicate that miRNA-99a acts as a suppressor in multiple tumors, including breast cancer. Overexpression of miRNA-99a exerts multiple inhibitory effects on breast cancer cells, reducing their vitality, inhibiting proliferation, and suppressing cell dissemination, thereby potentially slowing down tumor growth. On the other hand, miRNA-99a induces apoptosis in cancer cells, aiding in the clearance of abnormal cells, making miRNA-99a crucial for halting the development of breast cancer. MiRNA-99a may serve as a potential therapeutic target, contributing to improved patient survival rates.

The survival analysis conducted on breast cancer cases demonstrates that the miRNA predicted by the proposed model may play a significant role in early prevention, early diagnosis, and prognosis assessment of breast cancer. Additionally, this affirms the potential applicability of the proposed model in the biomedical field, holding promise for providing support in the research and treatment of breast cancer.

## Conclusion

It is crucial to comprehend the association between miRNAs and diseases, as abnormal expression of miRNAs can result in the occurrence of specific diseases. To improve accuracy and efficacy in predicting potential miRNA-disease associations, we have developed a new method called MFCAMNet. MFCAMNet uses an attention mechanism to weigh the multiple similarity matrices and an autoencoder to effectively reduce the dimensionality of the input features and generate low-dimensional miRNA and disease feature expressions. MFCAMNet then employs multiple strategies to generate miRNA-disease pair features. Finally, the Encoder Layer in Transformer was utilized to predict the association between miRNA and disease. In addition, we analyze the effect of various means of fusing feature strategies on performance and experimentally show that multiple source features can provide diverse information for deep learning models from different perspectives. By applying 5-fold and 10-fold cross-validation, MFCAMNet obtained the highest AUC and AUPR scores in comparison to other methods. In addition, we demonstrated the reliability of MFCAMNet with the outcomes of case studies involving three diseases.

However, the limitations of the proposed model must still be addressed. We construct multiple similarity matrices from multi-source data as the model input and then generate miRNA and disease pair features via element-level addition, multiplication, and concatenation. The questions that need to be discussed are how to effectively determine the most effective strategy for integrating the features and which fusion strategies have not been considered. In addition, many studies have employed miRNA functional similarity, disease semantic similarity, and Gaussian interaction profile similarity as supplemental information to predict the association between miRNAs and diseases. In practice, however, we found it challenging to guarantee that a large number of miRNAs and diseases have this information, making the solution to this problem a formidable obstacle.

## Data Availability

The code used in the current study is available at:https://github.com/yangchen0372/MFCAMNet.The datasets used in this study, HMDD v2.0 and HMDD v3.2, are available at: https://www.cuilab.cn/hmdd.
